# Suitability of Phytosterols Alongside Fatty Acids as Chemotaxonomic Biomarkers for Phytoplankton

**DOI:** 10.3389/fpls.2016.00212

**Published:** 2016-03-02

**Authors:** Sami J. Taipale, Minna Hiltunen, Kristiina Vuorio, Elina Peltomaa

**Affiliations:** ^1^Lammi Biological Station, University of HelsinkiLammi, Finland; ^2^Department of Environmental and Biological Sciences, University of Eastern FinlandJoensuu, Finland; ^3^Department of Biological and Environmental Science, University of JyväskyläJyväskylä, Finland; ^4^Finnish Environment Institute (SYKE)Jyväskylä, Finland; ^5^Department of Environmental Sciences, University of HelsinkiHelsinki, Finland

**Keywords:** freshwater, algae, biomolecules, lipids, chemotaxonomy, PERMANOVA, PERMDISP

## Abstract

The composition and abundance of phytoplankton is an important factor defining ecological status of marine and freshwater ecosystems. Chemotaxonomic markers (e.g., pigments and fatty acids) are needed for monitoring changes in a phytoplankton community and to know the nutritional quality of seston for herbivorous zooplankton. Here we investigated the suitability of sterols along with fatty acids as chemotaxonomic markers using multivariate statistics, by analyzing the sterol and fatty acid composition of 10 different phytoplankton classes including altogether 37 strains isolated from freshwater lakes. We were able to detect a total of 47 fatty acids and 29 sterols in our phytoplankton samples, which both differed statistically significantly between phytoplankton classes. Due to the high variation of fatty acid composition among Cyanophyceae, taxonomical differentiation increased when Cyanophyceae were excluded from statistical analysis. Sterol composition was more heterogeneous within class than fatty acids and did not improve separation of phytoplankton classes when used alongside fatty acids. However, we conclude that sterols can provide additional information on the abundance of specific genera within a class which can be generated by using fatty acids. For example, whereas high C_16_ ω-3 PUFA (polyunsaturated fatty acid) indicates the presence of Chlorophyceae, a simultaneous high amount of ergosterol could specify the presence of *Chlamydomonas* spp. (Chlorophyceae). Additionally, we found specific 4α-methyl sterols for distinct Dinophyceae genera, suggesting that 4α-methyl sterols can potentially separate freshwater dinoflagellates from each other.

## Introduction

The composition and abundance of phytoplankton is an important factor defining secondary and primary production, since phytoplankton synthesize many essential biomolecules (e.g., fatty acids, sterols, amino acids), which consumers cannot synthesize *de novo* (Harrison, 1990; Sargent et al., 1999; Lafont, 2000; Brett et al., 2009; Martin-Creuzburg and Von Elert, 2009). However, in aquatic ecology phytoplankton are often assumed to be a homogenous group and a uniform food source for higher trophic levels, primarily in studies using stable isotopes (Karlsson et al., 2003; Taipale et al., 2008; Solomon et al., 2011). The dietary quality of distinct phytoplankton classes for zooplankton is nowadays known to vary primarily due to the different availability of ω-3 and ω-6 fatty acids (Jonasdottir, 1994; Brett et al., 2009), but also because of lack of sterols (Von Elert et al., 2003). Compared to the laborious work of microscopic identification of phytoplankton, chemotaxonomic markers (e.g., pigments and fatty acids) provide a faster method for monitoring changes in a phytoplankton community (Mackey et al., 1996; Strandberg et al., 2015), but also provide information on the nutritional quality of seston for the whole food web. The use of sterols as chemotaxonomic biomarkers alongside fatty acids could potentially enhance separation of phytoplankton classes, but also provide information of the potential limitation of sterols in zooplankton diet. However, the similarity of sterol profiles among distinct phytoplankton classes is not yet analyzed using modern multivariate statistics, which have been previously used for fatty acids in freshwater phytoplankton, marine macrophytes, and macroalgae (Galloway et al., 2012; Kelly and Scheibling, 2012; Taipale et al., 2013).

Due to the great nutritional value and conservative transfer of fatty acids on higher trophic levels, fatty acids have been used as trophic markers (FATM, Fatty Acid Trophic Markers; Dalsgaard et al., 2003) for providing insight into consumer diets in pelagic and benthic food webs (Stott et al., 1997; Kelly and Scheibling, 2012). Fatty acid profiles of freshwater microalgae and marine macroalgae are mainly phylogeny-dependent, and environmental conditions influence the fatty acid composition only slightly (Galloway et al., 2012; Taipale et al., 2013; Galloway and Winder, 2015). Thus, a Bayesian mixing model based on fatty acids has been used successfully for describing phytoplankton community structure (Strandberg et al., 2015) as well as for taxonomic primary production (Dijkman et al., 2009). Non-metric multidimensional scaling analysis (NMS) of freshwater phytoplankton can separate green algae (Chlorophyceae, Trebouxiophyceae, Conjugatophyceae), diatoms (Diatomophyceae), and euglenoids (Euglenophcyeae) into their own groups, but clusters Cryptophyceae, Chrysophyceae, and Raphidophyceae in one group (Taipale et al., 2013). Additionally, fatty acid profiles of Cyanophyceae are highly variable (Los and Mironov, 2015), and thus chemotaxonomic markers for better phytoplankton phylogenetic separation are needed.

Sterols could give additional information on phytoplankton community structure, but also together with the essential ω-3 and ω-6 fatty acids on the biochemical quality of phytoplankton for aquatic food webs since both are essential for herbivorous consumers (Brett et al., 2009; Martin-Creuzburg and Von Elert, 2009). Phytosterols have been intensively studied since they were found in the 1930s (Carter et al., 1939; Patterson, 1991). But, perhaps due to the long research history of sterols, the same sterol can have several names in the literature, thus making sterol nomenclature confusing and complex. Phytosterols are sterols that are structurally similar to cholesterol but are synthesized in plants and phytoplankton. Usually, they are characterized by alkyl groups (methyl or ethyl group) at C-24, by side-chain double bonds at C-22 or C-24 or/and by nuclear double bonds at positions other than C-5 (Morreau et al., 2002). Among phytoplankton, the Dinophyceae are an exception because they can have a methyl group in the C-4 or C-23 position (Withers et al., 1978; Piretti et al., 1997). Some Chlorophyceae contain unusual Δ^7^-sterols such as chondrillasterol (Patterson, 1991; Thompson, 1996), whereas some Chlorophyceae in the order Chlamydomonadales have ergosterol (Gealt et al., 1981; Brumfield et al., 2010), which is also found in Euglenophyceae (Patterson, 1991). Full chemical names for sterols are included at Table 1. There is a great diversity of sterols in Diatomophyceae, but none of them are found exclusively in diatoms (Rampen et al., 2010). For example brassicasterol is also a major sterol in Cryptophyceae (Rampen et al., 2010) and stigmasterol, and β-sitosterol are typical for Chrysophyceae and Raphidophyceae in addition to Diatomophyceae (Cranwell et al., 1988; Rampen et al., 2010; Leblond et al., 2013).

**Table 1:** **Summary of the phytosterols analyzed from phytoplankton using the GC-MS method described in the methods section**.

**RT (min)**	**RRT**	**Systematic name**	**Trivial name**	**Formula**	**Position of double bond**	**C24-Alkylation**	**TMS M+**	**M+ - 15**	**Base peak**	**Other M+ ions**
15.9	1.0	5α-cholestane	Cholestane	C_27_H_48_			372	357	217	357, 262
19	1.19	Cholesta-5,24-dien-3β-ol	Desmosterol	C_27_H_44_O	Δ^5, 24(25)^		456	441	69	366, 351, 343, 327, 253, 129
19.7	1.24	Cholest-5-en-3β-ol	Cholesterol	C_27_H_46_O	Δ^5^		458	443	129	368, 353, 329, 255, 247, 228, 213
20	1.26	5α-cholestan-3β-ol	Epicholestanol	C_27_H_48_O			460	445	75	370, 369, 355, 306, 230, 148, 108, 106
20.3	1.28	(22E)-ergosta-5,22-dien-3β-ol	Brassicasterol or Diatomsterol	C_28_H_46_O	Δ^5, 22^	Methyl	470	455	69	381, 366, 341, 253, 251, 215, 213, 207
21.2	1.33	(22E)-ergosta-5,7,22-trien-3β-ol	Ergosterol	C_28_H_44_O	Δ^5, 7, 22^	Methyl	468	453	363	378, 376, 363, 362, 337, 253, 251, 237, 211
21.3	1.34	Ergosta-5,24(24^1^)-dien-3β-ol	24-Methylenecholesterol/ Ostreasterol/Chalinasterol	C_28_H_46_O	Δ^5, 24(28)^	Methylene	470	455	129	380, 371, 365, 343, 341, 296, 281, 253
21.4	1.35	Campest-5-en-3β-ol	Campesterol	C_28_H_48_O	Δ^5^	Methyl	472	457	129	382, 367, 343, 261, 255, 227, 213
21.5	1.35	(22E)-campesta-7,22-dien-3β-ol	Stellasterol	C_28_H_46_O	Δ^7, 22^	Methyl	470	455	69	343, 255, 213, 107
21.6	1.36	24-methyl-5α-cholest-24(28)-en-3β-ol	24-Methylcholestanol	C_28_H_48_O	Δ^24(28)^	Methylene	472	457	75	388, 345, 255, 215, 107
21.8	1.37	4-methyl-5α-cholestan-3β-ol	4-Methylcholestanol	C_28_H_50_O			474	459	75	459, 384, 369, 345
21.8	1.37	24-methyl-5α-cholestan-3β-ol	Campestanol	C_28_H_50_O		Methyl	474	459	215	459, 384, 369, 215
22	1.38	(24E)-stigmasta-5,24(24^1^)-dien-3β-ol	Fucosterol	C_29_H_48_O	Δ^5, 24(28)^	Ethylidene	484	469	55	386, 371, 343, 296, 281, 257, 129
22.1	1.39	(24E)-stigmasta-5,22-dien-3β-ol	Stigmasterol	C_29_H_48_O	Δ^5, 22^	Ethyl	484	469	83	394, 379, 355, 351, 255, 129
22.8	1.43	5α-ergost-7-en-3β-ol	Fungisterol	C_28_H_48_O	Δ^7^	Methyl	472	457	255	377, 367, 351, 255, 229, 213
22.8	1.43	3β-stigmasta-5,7,22-trien-3-ol	Corbisterol	C_29_H_46_O	Δ^5, 7, 22^	Ethyl	482	467	377	392, 351, 253, 211
23	1.45	Stigmasta-5-en-3β-ol	β-Sitosterol	C_29_H_50_O	Δ^5^	Ethyl	486	471	129	396, 381, 357, 255, 213
23.3	1.47	(22E)-5α-poriferasta-7, 22-dien-3β-ol	Chondrillasterol	C_29_H_48_O	Δ^7, 22^	Ethyl	484	469	55	441, 379, 372, 343, 318, 255
23.5	1.48	4α, 24-dimethyl-5α-cholestan-3β-ol		C_29_H_52_O		Methyl	488	473	75	473, 383, 359, 229
23.6	1.48	Unindetified C_30:2_ sterol		C_30_H_50_O			498	483	69	408, 368, 269, 139, 129, 83
23.7	1.49	4α,23,24-trimethyl-5α-cholest-22E-en-3β-ol	Dinosterol	C_30_H_52_O	Δ^22^	Methyl	500	485	69	359, 388, 271
23.7	1.49	5α-poriferast-7-en-3β-ol	22-Dihydrochondrillasterol	C_29_H_50_O	Δ^7^	Ethyl	486	471	486	471, 381, 255, 229, 213
23.8	1.50	Unidentified C_30:1_ sterol		C_30_H_52_O	?	?	500	485	69	359, 269, 95
24.5	1.54	4α,23,24-trimethyl-5α-cholest-24(28)-en-3β-ol		C_30_H_50_O	Δ^24(28)^	Methylene	500	485	95	387, 359, 297, 283
24.5	1.54	5α-stigmast-7-en-3β-ol	Schottenol	C_29_H_50_O	Δ^7^	Ethyl	486	471	255	396, 381, 345, 303, 230, 229, 213, 201
25.5	1.60	3β-gorgost-5-en-3β-ol	Gorgosterol	C_30_H_50_O	Δ^5^	Methyl	498	483	129	483, 408, 400, 386, 343, 337, 253
25.6	1.61	Unindetified C_30:1_ sterol		C_30_H_50_O	?	?	500	485	69	402, 373, 294, 373

*Relative retention time (RRT) was calculated in comparison to the retention time (RT) of cholestane (RRT = 1). The systematic name follows the IUPAC nomenclature and the trivial name is the most commonly used trivial name of the sterol. Position of the double bond refers to the carbon number which has a covalent double bond and C-24 alkylation shows what kind of alkyl group is bound to carbon number 24 (LIPID MAPS). Sterols were run as trimethylsilyl ethers (TMS) and were identified using specific ions, including the mass ion (TMS M^+^), and the most abundant mass ion (base peak; Jones et al., 1994)*.

Here we have studied the fatty acid and sterol profiles of 35 major freshwater phytoplankton genera (Tables 1, 2). Our 37 phytoplankton strains belong to 10 phytoplankton classes: Chlorophyceae, Conjugatophyceae, Cryptophyceae, Cyanophyceae, Diatomophyceae, Dinophyceae, Euglenophyceae, Raphidophyceae, Synurophyceae, and Trebouxiophyceae. We used multivariate statistics to describe the similarities and differences in fatty acid and sterol composition among freshwater phytoplankton classes and explored if the addition of sterols improves the differentiation between phytoplankton classes.

**Table 2:** **Summary of fatty acids analyzed from phytoplankton using the GC-MS method described in the methods section**.

**Fatty acid (C:D:ω)**	**RT (min)**	**Ion (*m/z*) for quantification**	**Reference ions (*m/z*)**
13:0	9.365	74	228, 87, 69
13:1	9.8	74	228, 87, 69
i-14:0	11.4	74	87, 199, 242
14:0	11.445	74	87, 143, 242
14:1	11.9	74	87, 143, 242
i-15:0	13.7	74	87, 43, 256
a-15:0	13.9	74	87, 256, 55
15:0	14.215	74	87, 143, 256
15:1ω7	14.9	74	55, 69, 254
i-16:0	16.2	74	87, 270, 143
16:0	17.69	74	87, 143, 270
16:1ω9	18.2	74	55, 69, 268
16:1ω7t	18.4	74	69, 268
16:1ω8c	18.7	74	69, 268
16:1ω7c	18.65	74	69, 268
16:1ω6c	19.1	74	69, 268
16:1ω5t	19.3	74	69, 268
16:1ω5c	19.4	74	69, 268
16:2ω6	19.7	67	74, 266, 69
i-17:0	21.7	74	284, 143, 57
a-17:0	21.9	74	284, 143, 57
16:2ω5	22	67	74, 69, 266
16:2ω4	22.1	67	74, 69, 266
17:0	21.81	74	87, 55, 284
16:3ω4	22.5	79	122, 194, 264
16:3ω3	22.6	79	108, 208, 264
17:1ω9c	22.96	74	55, 282, 69
17:1ω7c	23.6	74	69, 282, 83
16:4ω3	23.8	79	166, 108
16:4ω1	24	79	266, 80, 194
17:2	24.1	67	280, 55
18:0	26.4	74	87, 298
Cy-19:0t	27.4	74	69, 296, 56
18:1ω9t	27.9	74	69, 296, 56
18:1ω9c	27.5	74	69, 83, 296
18:1ω8c	28.2	74	69, 296, 83
18:1ω7c	28	74	69, 296, 83
18:1ω6c	28.7	74	69, 296, 83
18:2ω7,12c	29.3	67	294, 69, 55
18:2ω6t	29.785	67	81, 95, 294
18:2ω6c	30	67	294, 69, 55
18:3ω6	31.105	79	292, 150, 194
18:3ω4	32	79	122, 222, 292
19:0	32.2	74	87, 312, 143
18:3ω3	32.95	79	236, 108, 292
18:4ω3	33.5	79	108, 194
18:5ω3	35	79	108, 152
20:0	37.4	74	87, 326
20:1ω9	38	74	69, 324
20:1ω7	39.2	74	69, 324
20:2ω6	40.8	67	322, 81
20:3ω6	42	79	150, 320, 222
20:4ω6	43	79	150, 180, 318
20:3ω3	44.33	79	320, 264, 108
20:4ω3	46	79	108, 222, 318
20:5ω3	46.75	79	108, 180, 316
22:0	49	74	87, 143, 354
22:1ω9c	50	69	55, 69, 320
22:1ω7c	50.4	69	55, 74, 320
22:2ω6	52.5	67	350, 55
21:5ω3	53	79	108, 194
23:0	53.4	74	368, 87
22:3ω6	54.7	79	150
22:4ω6	54.2	79	366, 150, 313
22:5ω6	54.24	79	150, 166
22:5ω3	56.7	79	108, 208
22:6ω3	57	79	108, 166
24:0	57.5	74	87, 382, 143
24:1	58	69	348, 380
26:0	60	74	87, 382, 143
28:0	62	74	87, 438, 143

*Fatty acids are presented in the order they eluted (RT, retention time in minutes) from the DB-23 column. Reference ion was used for fatty acid identification and major peak for quantification. Names for fatty acids are presented as C:D:ω; C, the number of carbon in the chain; D, the number of double bonds; ω, position of first double bond. Additionally, c and t cites refer to cis or trans transfiguration. Abbreviation i- (iso) and a- (anteiso) cites to fatty acids with methyl branched position at second or third carbon from the end. Cy- cites to the cyclopropane fatty acid*.

## Materials and methods

### Phytoplankton culturing

The algal classification follows the taxonomy and trivial names of AlgaeBase (Guiry and Guiry, 2014). Class, order, and species of the cultured strains are presented in Table 3. The cultured phytoplankton genera were chosen based on the most abundant taxa in the phytoplankton database of the Finnish Environment Institute (SYKE). The phytoplankton data represent on average 81% of the total phytoplankton biomass in Finnish lakes during open water season (June–September, in 1975–2013), and they also represent the most abundant phytoplankton genera in lakes across the boreal zone, including Northern Great Britain, Norway, Sweden, and Finland (Järvinen et al., 2013; Maileht et al., 2013). Phytoplankton composition varies in boreal lakes by total phosphorus (μg l^−1^), water color (mg l^−1^ Pt), and Secchi-disc transparency (Lepistö and Rosenström, 1998).

**Table 3:** **Class, order, species, and the strain code information of the studied freshwater phytoplankton**.

**Class**	**Order**	**Species**	**Number here**	**Strain**	**Media**	**Light cycle**	**Light intensity**	**Temperature**
Cyanophyceae	Chroococcales	*Aphanothece cf. clathrata*	1	NIVA-CYA 369	MWC^1^	14:10	50	20
(cyanobacteria)	Chroococcales	*Microcystis* sp.	2	NIVA-CYA 642	MWC^1^	14:10	50	20
	Chroococcales	*Snowella lacustris*	3	NIVA-CYA 339	MWC^1^	14:10	50	20
	Synechococcales	*Synechococcus elongatus*	4	UTEX LB 563	MWC^1^	14:10	50	20
	Nostocales	*Anabaena flos-aquae*	5	NIVA 138	MWC^1^	14:10	50	20
	Oscillatoriales	*Phormidium tenue*	6	NIVA-CYA 25	MWC^1^	14:10	50	20
	Oscillatoriales	*Planktothrix rubescens*	7	SCCAP K-576	MWC^1^	14:10	50	20
	Pseudanabaenales	*Limnothrix planktonica*	8	NIVA-CYA 107	MWC^1^	14:10	50	20
	Pseudanabaenales	*Pseudanabaena limnetica*	9	NIVA 276/11	MWC^1^	14:10	50	20
	Pseudanabaenales	*Pseudanabaena* sp.	10	SCCAP K-1230	MWC^1^	14:10	50	20
Cryptophyceae	Cryptomonadales	*Cryptomonas marssonii*	11	CCAP 979/70	DY-V^2^	16:8	30	20
(cryptomonads)	Cryptomonadales	*Cryptomonas ovata*	12	SCCAP K-1876	AF6^3^	16:8	30	20
	Pyrenomonadales	*Rhodomonas minuta*	13	CPCC^7^ 344	L16^4^	14:10	30	18
Dinophyceae	Gonyaulacales	*Ceratium*	14	Lake Köyhälampi	Lake Köyhälampi	–	–	15
(dinoflagellates)	Peridiniales	*Peridinium cintum* (n=3)	15	SCCAP K-1721	MWC^1^	14:10	70	20
Synurophyceae	Synurales	*Mallomonas caudata*	16	Lake Horkkajärvi	Lake Horkkajärvi	–	–	20
(golden algae)	Synurales	*Synura* sp.	17	SCCAP K-1875	MWC^1^	16:8	30	20
Raphidophyceae	Chattonellales	*Gonyostomum semen*	18	LI21	MWC^1^	16:8	80	20
Diatomophyceae	Tabellariales	*Tabellaria* sp.	19	CCAP 1081/7	Chu 10 ^5^	14:10	40	18
(diatoms)	Tabellariales	*Tabellaria* sp.	20	CCAP 1081/7	Z8^6^	14:10	40	18
	Aulacoseirales	*Aulacoseira ex Melosira granulata*	21	CPCC 397	Chu 10 ^5^	14:10	40	18
	Thalassiosirales	*Cyclotella meneghiniana*	22	CCAC 0039	MWC^1^	14:10	40	18
	Fragilariales	*Asterionella formosa*	23	NIVA-BAC-3	MWC^1^	14:10	40	18
	Fragilariales	*Fragilaria crotonensis*	24	UTEX LB FD56	MWC^1^	14:10	40	18
	Fragilariales	*Diatoma tenuis*	25	CPCC 62	Chu 10 ^5^	14:10	40	18
	Fragilariales	*Synedra rumpens* var. *familiaris*	26	NIVA-BAC 18	MWC^1^	14:10	40	18
Euglenophyceae	Euglenales	*Euglena gracilis*	27	CCAP^3^ 1224/5Z	AF6^3^	16:8	40	20
(euglenoids)	Euglenales	*Euglena gracilis*	28	CCAP^3^ 1224/5Z	EG^7^	16:8	40	20
Chlorophyceae	Chlamydomonadales	*Sphaerocystis* sp.	29	Lake Majajärvi	Lake Majajärvi			20
(green algae)	Chlamydomonadales	*Eudorina* sp.	30	K-1771	MWC^1^	14:10	70	20
	Chlamydomonadales	*Chlamydomonas reindhardtii*	31	UWCC	MWC^1^	14:10	70	20
	Sphaeropleales	*Monoraphidium griffithii*	32	NIVA-CHL 8	MWC^1^	14:10	70	20
	Sphaeropleales	*Pediastrum* sp.	33	SCCAP K-1033	MWC^1^	14:10	70	20
	Sphaeropleales	*Acutodesmus* sp.	34	University of Basel	MWC^1^	14:10	70	20
	Sphaeropleales	*Selenastrum* sp.	35	SCCAP K-1877	MWC^1^	16:8	70	20
Trebouxiophyceae	Prasiolales	*Botryococcus* sp.	36	SCCAP K-1033	MWC^1^	14:10	70	20
Conjugatophyceae	Desmidiales	*Closterium* sp.	37	CPCC 288	Z8^6^	14:10	50	18
	Desmidiales	*Cosmarium reniforme*	38	SCCAP K-1145	MWC^1^	14:10	50	18
	Desmidiales	*Staurastrum* sp.	39	SCCAP K-1349	MWC^1^	14:10	50	18

*Different media and light cycle, i.e., the light:dark period (h), light intensity (μmol m^−2^ s^−1^), and temperature (°C) were used for different strains. Strains 14, 16, and 29 were not cultured, but collected form lakes during their blooms. All the strains are numbered and the same numbers are used in **Figure 2** Green algae include classes Chlorophyceae, Trebouxiophyceae, and Conjugatophyceae*.

1 *Guillard and Lorenzen, 1972; Guillard, 1975*;

2 *Andersen et al., 1997*;

3 *Watanabe et al., 2000*;

4 *Lindström, 1983*;

5 *Chu, 1942*;

6 *Staub, 1961; Kótai, 1972; ^7^UTEX*.

Freshwater strains of some abundant taxa (e.g., Cyanophyceae *Woronichinia* and Dictyocophyceae *Pseudopedinella*) in boreal freshwaters were not available in any culture collection. Moreover, some strains, e.g., *Urosolenia* (Diatomophyceae) and *Dinobryon* (Chrysophyceae), grew extremely slowly, and thus we were not able to obtain enough biomass for sterol analysis. Additionally, *Ceratium* (strain 14), *Mallomonas* (strain 16), and *Sphaerocystis* (strain 29) were not cultured, but collected form lakes during their blooms.

Most of the phytoplankton strains were grown at 18°C under a 14:10 h light:dark cycle with light intensity of 30–70 μmol m^−2^ s^−1^. Each strain was cultured in a medium specific to that strain (Table 3). Additionally, *Tabellaria* (strain 19 and 20) was cultured in two different media (Chu10 and Z8) and *Euglena gracilis* (strain 27 and 28) with (EG) and without (AF6) organic substrates (Table 3). The strains of *Cryptomonas marssonii* (strain 11), *Cryptomonas ovata* (strain 12), *Synura* sp. (strain 17), *Gonyostomum semen* (strain 18), *E. gracilis* (strain 27 and 28), and *Selenastrum* sp. (strain 35) were cultured at 20°C with light instensity of 40 μmol m^−2^ s^−1^ and using a 16:8 h light:dark cycle. We used plastic or glass flasks, volume > 200 ml. Depending on the cell density, 1–6 ml of the phytoplankton stock was inoculated per 200 ml of fresh culture media every 2 weeks. The samples for phytoplankton analyses were harvested in the late phase of exponential growth, i.e., 2–3 weeks after the inoculation.

### Fatty acid analyses

Lipids from freeze-dried, homogenized phytoplankton (1–3 mg) were extracted using chloroform: methanol: NaCl mixture with volumes of 2, 1, 0.75 mL of chloroform, methanol, and 2% NaCl as reported in Parrish (1999). Phytoplankton samples were sonicated for 10 min and vortexed (2–3X) and the lower organic phases were removed after which 2 mL of chloroform was added. After centrifuging the lower organic phase was removed and pooled, and the organic solvent was evaporated to dryness. For the formation of fatty acid methyl esters (FAME), 1 mL of toluene, and a 2 mL of sulphuric acid-methanol solution were used with dried lipids (all of each sample), and incubated at 80°C in a water bath for 2 h. Samples were neutralized by adding 2 mL of 2% KHCO_3_ and FAMEs diluted in 5 mL of hexane. The upper layer was transferred into a clean centrifuge tube after which this step was repeated, and finally hexane was evaporated to dryness under nitrogen flow and the sample was transferred to autosampler vials with inserts (Agilent, Santa Clara, United States) with 200 μL hexane.

FAMEs were analyzed with a gas chromatograph (Shimadzu Ultra, Kyoto, Japan) equipped with mass detector (GC-MS) and using helium as a carrier gas. An Agilent® (Santa Clara, California, U.S.A.) DB-23 column (30 m × 0.25 mm × 0.15 μm) was used with the following temperature program: 60°C was maintained for 1.5 min, then the temperature was increased at 10°C min^−1^ to 100°C, followed by 2°C min^−1^ at 140°C, and 1°C min^−1^ at 180°C and finally heated at 2°C min^−1^ to 210°C and held for 6 min. Fatty acids were identified by the retention times (RT) and using specific ions (Table 2). Fatty acid concentrations were calculated using calibration curves based on known standard solutions of a FAME standard mixture (GLC standard mixture 566c, Nu-Chek Prep, Elysian, Minnesota, U.S.A.). The Pearson correlation coefficient was >0.99 for each individual fatty acid calibration curve (details of the method in Taipale et al., 2013).

### Sterol analyses

Replicates of dried total lipid samples (subsamples of the ones used for fatty acids) of phytoplankton were used for sterol analysis. To separate sterols from fatty acids, lipid samples of cultured strains were saponified by adding 2 mL of 1.2 N methanolic KOH and samples was kept at 70°C for 2 h (in water bath). After this saponification, 1 mL of 2% sodium chloride and 3 mL of hexane were added to the samples, and they were vortexed and centrifuged. The upper organic layer was transferred to a new acetone-washed tube (precombusted at 440°C for 4 h) and 3 mL of hexane were added, and then this step was repeated to maximize the yield of the non-polar phase. After evaporating hexane to dryness, samples were dried with acetone for removing any residues of water or moisture and to maximize the silylation reaction in the next stage.

For silylation, 0.2 mL of *N,O-bis*[trimethylsilyltrifluoro-acetamide] (BSTFA) with 1% (w) trimethylchlorosilane (TMCS) (Fluka Sigma-Aldrich, St. Louis, Missouri, U.S.A.) and 0.2 mL of pyridine (Sigma-Aldrich, St. Louis, Missouri, U.S.A.) were added to the samples and incubated for 2 h at 70°C, after which the samples were evaporated and transferred to glass vials with inserts. The samples were dissolved in 50–200 μl of hexane. Trimethylsilyl (TMS) derivatives of sterols were analyzed with a gas chromatograph (Shimadzu) equipped with a mass detector. A Phenomenex® (Torrance, California, U.S.A.) ZB-5 Guardian column (30 m × 0.25 mm × 0.25 μm) was used with the following temperature program: 150°C was maintained for 1 min, then the temperature was increased at 15°C min^−1^ to 280°C, finally heated by 2°C min^−1^ to 320°C, and held for 10.3 min. The total length of the temperature program was 40 min. Retention times and relative retention times (RRT) for detected sterols are presented in Table 1. Helium was used as a carrier gas with average velocity of 34 cm sec^−1^. Sterols were quantified using authentic standard solutions of plant sterol mixture from Larodan (Solna, Sweden; including 53% β-sitosterol, 7% stigmasterol, 26% of campesterol, 13% of brassicasterol), and cholesterol and fucosterol from Sigma-Aldrich. Sterol concentrations were calculated using four point calibration curves based on known standard concentrations (between 1.3 and 159 μg/μL). The Pearson correlation coefficient was >0.99 for each individual sterol calibration curve. Recovery percentage of sterol samples was calculated using 5-α-cholestane (Sigma-Aldrich) as an internal standard.

The identification of TMS ethers of sterols was based on the standard mixes described above, retention times together with the NIST Mass Spectral Database 11 (http://chemdata.nist.gov) and literature (Rahier and Benveniste, 1989; Jones et al., 1994; Goad and Akhisa, 1997). The molecular ion peak, molecular ion −15 (M+-15), base peak and other used ions for identification are presented in Table 1.

### Statistical analyses

We used non-metric multidimensional scaling (NMS) to visualize the variation in fatty acid and sterol composition and in a combined dataset of sterols and fatty acids (contribution of each sterol and fatty acids was calculated from the sum concentration of all sterols and fatty acids) among phytoplankton classes. Permutational multivariate analysis of variance (PERMANOVA, Anderson et al., 2008) was used to test whether these differences in the sterol and/or fatty acid composition were statistically significant. PERMANOVA was run with unrestricted permutation of raw data and type III sums of squares. Similarity percentages (SIMPER) were used to identify the characteristic sterols and fatty acids of each phytoplankton class. We used PERMDISP (Anderson, 2006) to investigate the within-class variation in fatty acid and/or sterol composition. All the multivariate analyses were operated on Euclidean distances of untransformed data with the program PRIMER-E (v.6; Ivybridge, United Kingdom) and the PERMANOVA+ add-on.

## Results

### Phylogenetic variation in sterol and fatty acid profiles

**Figure 1:**
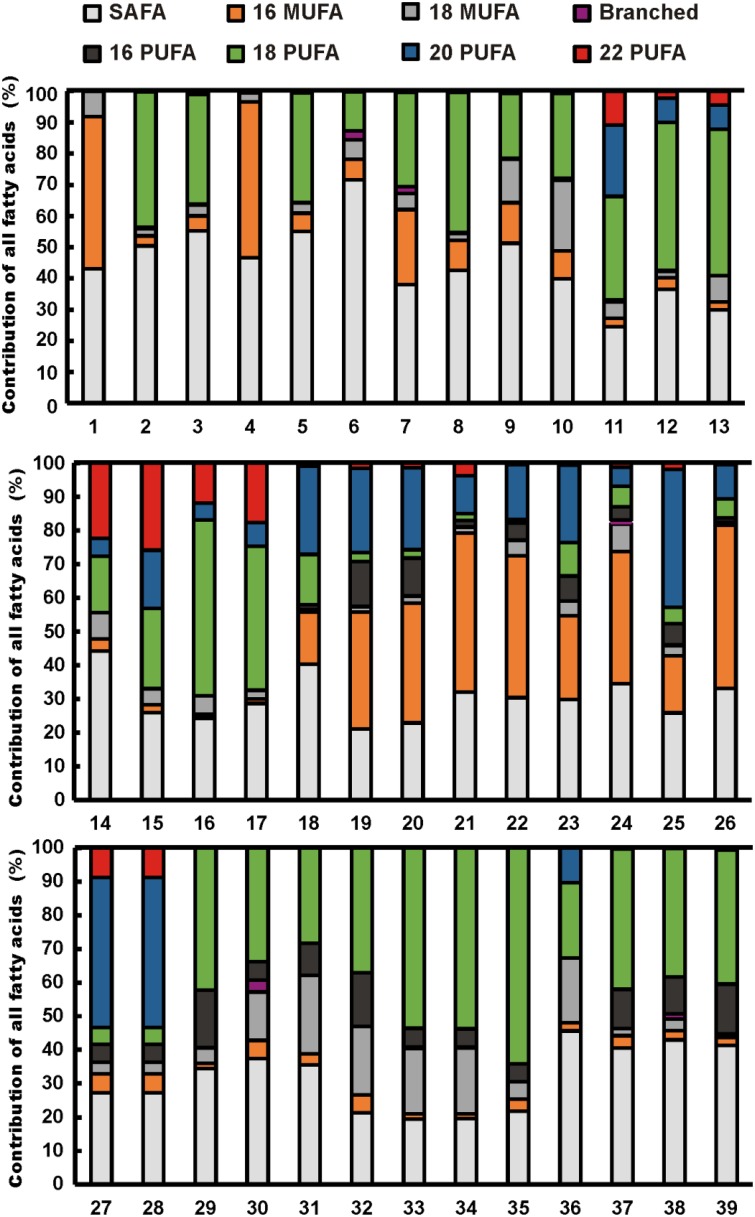
**Fatty acid profiles of cultured freshwater phytoplankton strains**. Strain numbers are given in Table 2. Fatty acids are presented as major groups: saturated fatty acids (SAFA), C_**16**_ and C_**18**_ monounsaturated fatty acids (C_**16**_ MUFA, C_**18**_ MUFA), branched fatty acids, and C_**16**_, C_**18**_, C_**20**_, and C_**22**_ polyunsaturated fatty acids (PUFA).

**Figure 2:**
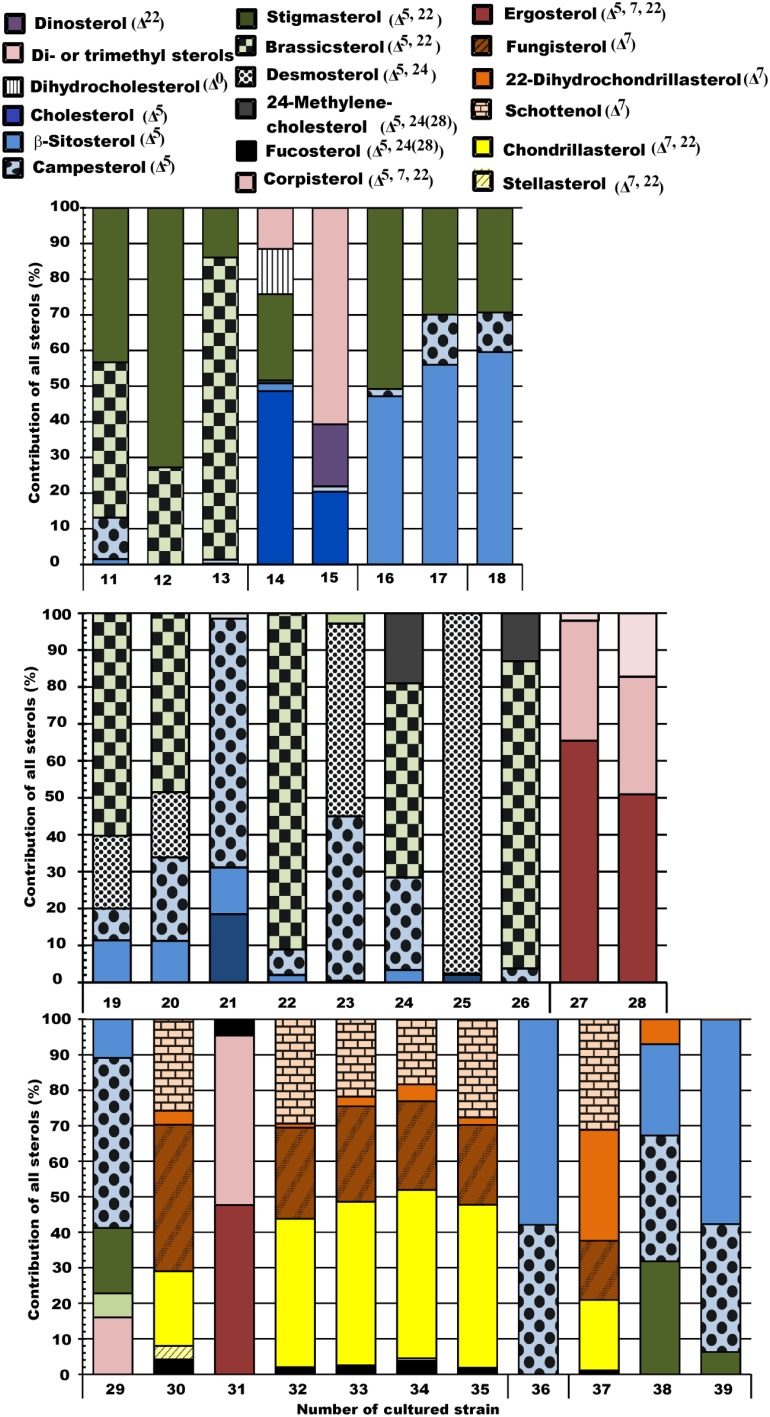
**Sterol profiles of the studied phytoplankton strains**. Orange-brown represents Δ^7^ unsaturation, yellow represents Δ^7, 22^ diunsaturation, blue represents Δ^5^ unsaturation, green represents Δ^5, 22^ diunsaturation, black represents ^Δ5, 24(28)^ diunsaturation, red represents Δ^5, 7, 22^ triunsaturation, and violet represents 4α-methyl and 23,24 -trimethyl sterols.

We were able to detect 44 fatty acids and 29 sterols (Tables 1, 2) in the studied freshwater algal strains (Figures 1, 2). The fatty acid and sterol profiles of the eight freshwater phytoplankton classes (excluding Raphidophyceae, which included only one strain) differed significantly from each other. Class identity explained 52% of variation in the algal fatty acids [PERMANOVA, *F*_(9, 29)_ = 4.59, *p* = 0.0002] when Cyanophyceae were included and 71% when Cyanophyceae were excluded [PERMANOVA, *F*_(8, 20)_ = 8.28, *p* = 0.0002]. Respectively, class identity explained 51% of the variation in sterol profiles when Cyanophyceae were included [PERMANOVA, *F*_(9, 29)_ = 4.65, *p* = 0.002] and 42% when Cyanophyceae, which had no sterols, were excluded [PERMANOVA, *F*_(8, 20)_ = 3.20, *p* = 0.0002]. The explained variation by the factor “class” was 70% [PERMANOVA, *F*_(9, 29)_ = 4.59, *p* = 0.0002] and 50% [PERMANOVA, *F*_(9, 29)_ = 7.84, *p* = 0.001] when the fatty acid and sterol datasets were combined and when Cyanophyceae were excluded or included, respectively.

According to the *post-hoc* pairwise test, the combined dataset of fatty acids and sterols differentiated between the same classes as the fatty acids alone (Table 4). Those phytoplankton classes that could not be separated with fatty acids or with the combined dataset of fatty acids and sterols were Cyanophyceae and Cryptophyceae, Cyanophyceae and Conjugatophyceae, and also Conjugatophyceae and Chlorophyceae. Due to the lack of sterols in Cyanophyceae, their sterol profile differed statistically from all other phytoplankton classes.

**Table 4:** **Monte Carlo *p*-values from the pair-wise comparisons (PERMANOVA) of fatty acid (FA), sterol (STE), fatty acid and sterol (FA+STE) composition**.

**Data**	**% explained by class**	**Cya, Cry**	**Cya, Dia**	**Cya, Chlo**	**Cya, Con**	**Cry, Dia**	**Cry, Chlo**	**Cry, Con**	**Dia, Chlo**	**Dia, Con**	**Chlo, Con**
**FA**	51.5	0.08	0.00^*^	0.01^*^	0.21	0.00^*^	0.03^*^	0.02^*^	0.00^*^	0.00^*^	0.07
**STE**	50.1	0.00^*^	0.00^*^	0.00^*^	0.00^*^	0.12	0.00^*^	0.05	0.00^*^	0.08	0.12
**FA**+**STE**	49.7	0.07	0.00^*^	0.01^*^	0.26	0.00^*^	0.02^*^	0.03^*^	0.00^*^	0.00^*^	0.09

*Statistically significant differences between two classes (Cya, Cyanophyceae; Cry, Cryptophyceae; Dia, Diatomophyceae; Chlo, Chlorophyceae; Con, Conjugatophyceae) are marked with stars. The factor Class explained most of variation when only fatty acids were included into analyses*.

**Figure 3:**
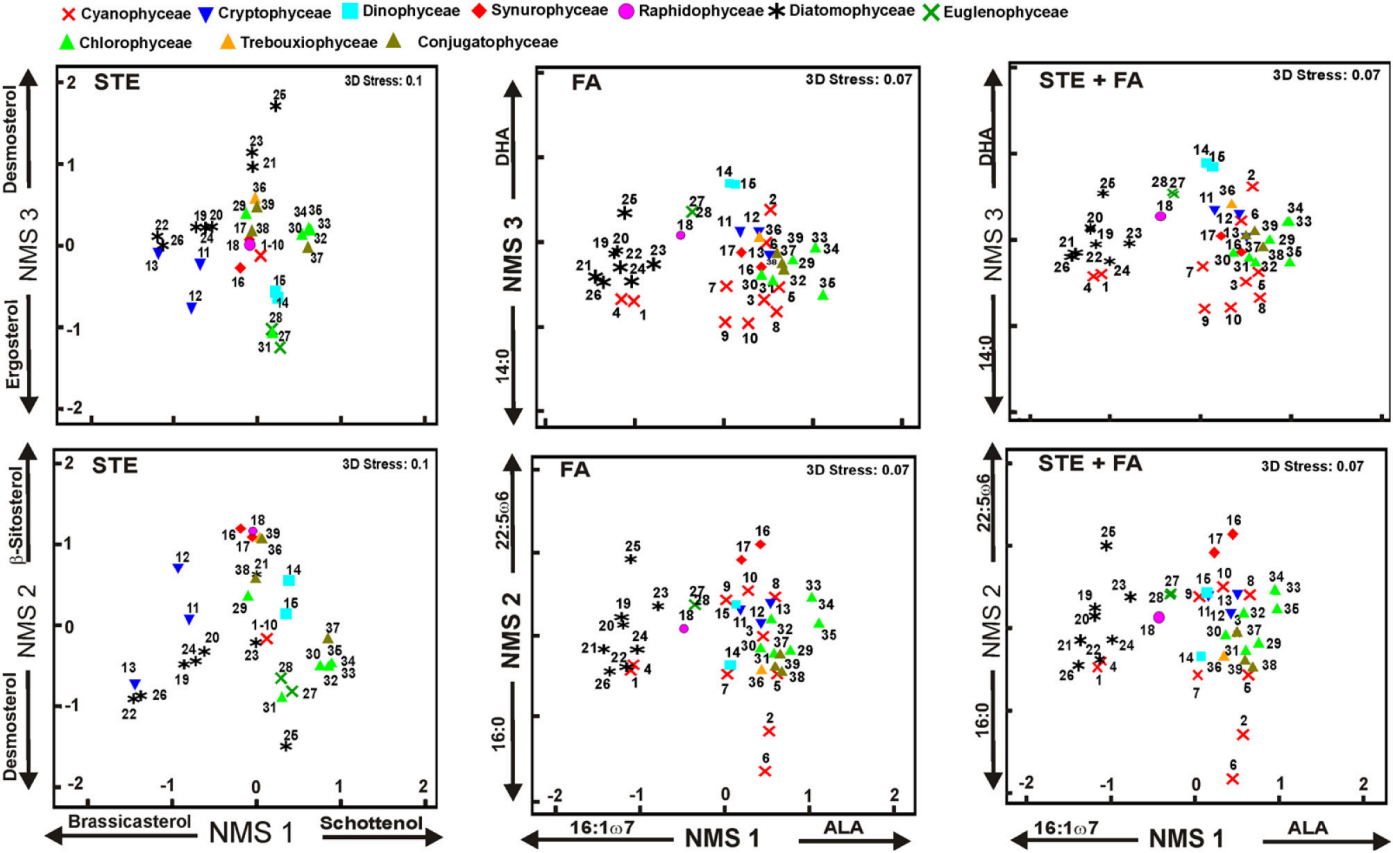
**Results of non-metric multidimensional scaling (NMS)**. The final stress was 0.1 for sterols and 0.07 for other analysis, indicating a reasonable ordination in three dimensions. The plot shows the similarity of ordination based on the sterol (STE), fatty acids (FA), and combined profiles (STE + FA). The strongest correlation of individual sterol or fatty acid is presented for all axes. Phytoplankton strains are presented in Table 3. Detailed list of correlations is presented in **Table 6**.

**Figure 4:**
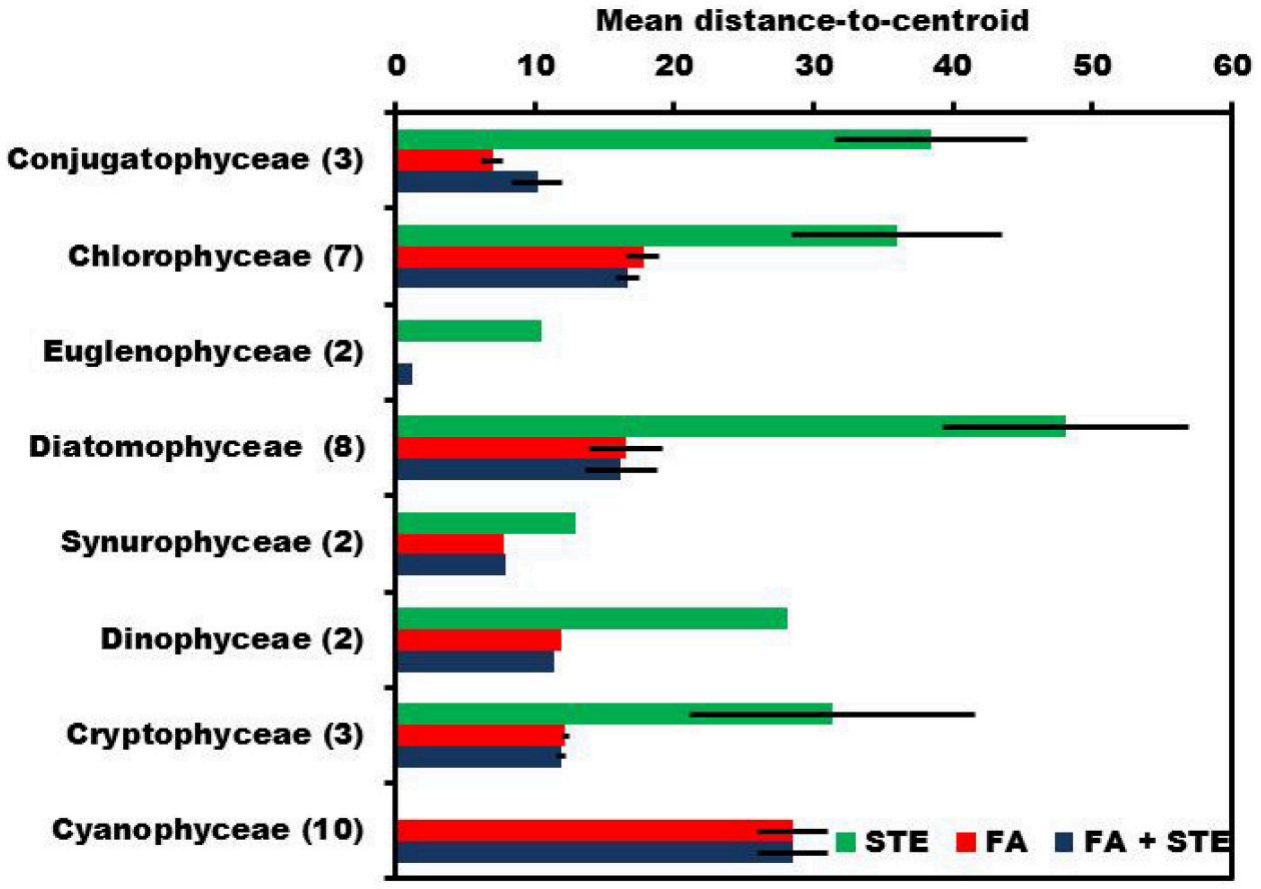
**PERMIDISP was used to evaluate dispersion within each phytoplankton class**. Highest mean distance-to-centroid was measured when sterols (STE) were used alone, but fatty acids (FA) and combined data of sterols and fatty acids (STE + FA) have similar dispersion.

The within-class multivariate dispersion varied between different phytoplankton classes in sterol, fatty acid, and combined signature datasets (Figure 3). Generally, dispersion varied among classes and the variation within phytoplankton class was higher in sterol signatures [PERMDISP; *F*_(9, 29)_=5.862] than in fatty acids [PERMDISP; *F*_(9, 29)_=9.6037] or in combined signatures [PERMDISP; *F*_(9, 29)_=9.131] (Figure 4). Sterol signatures were most similar in the Synurophyceae and Euglenophyceae, otherwise the mean distance-to-centroid in sterol signatures was two or even three times higher than in fatty acid signatures. The similarity of class was equal when only fatty acid data was used and also in the combined dataset of fatty acids and sterols, but in some cases sterols increased the integrity of the class (e.g., in Diatomophyceae).

Five sterols and fatty acids that contributed most to within-group (phytoplankton class) similarity and their mean proportions were identified with the SIMPER analysis (Table 5). The “Contributions” are the percent contributions of sterols or fatty acids to the similarities within the taxa in that class. When SIMPER analysis was performed with the combined datasets of sterols and fatty acids, only the classes Synurophyceae, Euglenophyceae, and Conjugatophyceae had sterols among the five most important classifying contributors. Sterols and fatty acids were most responsible for within-group similarities and also played an important role in separating the phytoplankton groups in the NMS (Figure 3).

**Table 5:** **Results of SIMPER analysis showing the five most important fatty acids (FA) and sterols (STE) contributing (Contrib%) to similarities within phytoplankton classes**.

**Algal Class**	**FA**	**Mean**	**Contrib%**	**STE**	**Mean**	**Contrib%**	**FA + STE**	**Mean**	**Contrib%**
Cyanophyceae (10)	16:0	31.9	29.0				16:0	31.9	29.0
	16:1ω7	13.3	26.3				16:1ω7	13.3	26.3
	ALA	18.3	17.4				ALA	18.3	17.4
	14:0	14.0	12.2				14:0	14.0	12.2
	18:3ω6	3.0	8.0				18:3ω6	3.0	8.0
Cryptophyceae (3)	EPA	12.1	27.6	Brassicasterol	51.8	49.2	SDA	14.5	22.4
	SDA	15.9	20.9	Stigmasterol	43.4	48.5	EPA	10.6	18.4
	ALA	23.5	15.8	Campesterol	4.2	2.3	16:0	18.5	12.6
	18:0	4.8	13.1				ALA	21.1	16.3
	16:0	20.6	11.1				18:0	4.4	11.7
Dinophyceae (2)	16:0	28.8	49.7	Cholesterol	34.5	25.1	16:0	27.1	48.9
	EPA	11.3	25.1	C_30:2_ sterol	14.1	25.1	EPA	10.6	24.5
	ALA	6.8	18.5	Stigmasterol	12.2	18.5	DHA	22.8	2.1
	DHA	24.1	2.2	4, 24-Dimethyl-5-cholestan-3-ol	9.2	10.8			
	18:1ω9	5.7	2.0	Dinosterol	8.7	9.5			
									
Synurophyceae (2)	ALA	18.5	42.9	Stigmasterol	40.4	66.1	ALA	17.9	45.1
	22:5ω6	8.6	28.7	Campesterol	8.1	22.0	22:5ω6	8.1	22.9
				β-Sitosterol	51.6	11.8	SDA	21.5	6.8
							16:0	6.6	9.5
							β-Sitosterol	2.2	3.1
Diatomophyceae (7)	EPA	16.1	41.6	Brassicasterol	42.3	42.2	EPA	15.7	41.0
	16:1ω7	36.1	31.4	Desmosterol	23.6	37.2	16:1ω7	35.1	31.0
	16:0	16.3	10.6	Campesterol	22.3	16.6	16:0	15.8	10.5
	14:0	10.2	5.6	24-Methylene-cholesterol	4.0	1.8	14:0	9.9	5.6
	ARA	3.4	2.4	Cholesterol	2.6	1.3	ARA	3.3	2.4
Euglenophyceae (2)	14:0	6.0	100.0	Unidentified C_30:1_ sterol	9.6	52.0	Unidentified C_30:1_ sterol	1.0	52.8
	20:0	0.1	0.0	Ergosterol	58.2	48.0	Corpisterol	2.9	19.6
				Corpisterol	32.2	0.1	Ergosterol	5.1	7.7
							16:0	15.4	6.0
							EPA	14.8	5.5
Chlorophyceae (7)	LIN	10.6	22.1	Chondrillasterol	28.9	24.6	LIN	9.6	20.6
	ALA	28.5	17.9	Campesterol	6.8	17.1	18:1ω7	7.4	18.7
	18:1ω7	7.9	17.2	Corpisterol	9.1	17.0	16:0	21.2	16.5
	18:1ω9	7.3	15.1	Ergosterol	6.8	17.0	18:1ω9	6.7	14.1
	16:0	23.1	14.2	Fungisterol	20.2	11.9	ALA	25.7	10.3
Conjugatophyceae (3)	16:4ω3	2.5	26.2	β-Sitosterol	27.8	35.4	16:0	32.5	27.5
	16:3ω3	8.8	26.2	Campesterol	23.8	18.0	16:3ω3	8.2	13.6
	ALA	27.8	15.7	Schottenol	10.4	13.7	Schottenol	2.6	12.7
	SDA	6.4	10.4	Stigmasterol	12.7	12.0	ALA	2.7	12.5
	16:0	36.1	9.1	22-Dihydro-chondrillasterol	12.8	11.3	22-Dihydro-chondrillasterol	24.7	8.3

*Mean shows average % of individual lipid biomolecule. The number of strains within one class is presented in parentheses after the class name. ALA, α-linolenic acid, 18:3ω3; SDA, stearidonic acid, 18:4ω3; EPA, eicosapentaenoic acid, 20:5ω3; DHA, docosahexaenoic acid, 22:6ω3; LIN, linoleic fatty acid, 18:2ω6; ARA, arachidonic acid, 20:4ω6)*.

NMS 1 of sterol data (Figure 3) was positively correlated (*p* < 0.05, *r* > 0.51) with Δ^7^ -sterols (schottenol, fungisterol), Δ^7, 22^ -sterols (chondrillasterol), and Δ^5, 24(28)^ -sterols (fucosterol, see all correlations in Table 6), which were the most abundant sterols in Chlorophyceae (*Acutodesmus, Eudorina, Monoraphidium, Pediastrum, Selenastrum*) and in one of the Conjugatophyceae (*Closterium*) (Table 4). NMS 1 was negatively correlated (*p* < 0.05, *r* = −0.87) with brassicasterol (Δ^5, 22^ sterol), which was common in all of the cultured Cryptophyceae (*Cryptomonas* and *Rhodomonas*) and in most of the Diatomophyceae (*Cyclotella, Fragilaria, Synedra, Tabellaria*). NMS 2 correlated positively (*p* < 0.05, *r* > −0.51) with stigmasterol (Δ^5, 22^ -sterol), campesterol, and β-sitosterol (Δ^5^ -sterol). Stigmasterol was abundant among the Cryptophyceae, Synurophyceae, and Dinophyceae. NMS 3 correlated positively (*p* < 0.05, *r* > 0.72) with desmosterol (Δ^5, 24^ -sterol), which was found in four strains of Diatomophyceae (*Asterionella, Aulacoseira, Diatoma, Tabellaria*), and was the major sterol of *Diatoma* (number 25 in Figures 1, 4). NMS 3 correlated negatively (*p* < 0.05, *r* = 0.6) with Δ^5, 7, 22^ –sterols including corbisterol and ergosterol which are abundant sterols in *Chlamydomonas* (Chlorophyceae) and *Euglena* (Euglenophyceae).

**Table 6:** **Pearson correlations between individual fatty acids and sterols and the NMS axes 1, 2, and 3**.

**Compound**	**Sterols**			**Fatty acids**			**Fatty acids** + **Sterols**		
	**NMS 1**	**NMS 2**	**NMS 3**	**NMS 1**	**NMS 2**	**NMS 3**	**NMS 1**	**NMS 2**	**NMS 3**
Cholesterol	0.13	0.21	−0.12				−0.07	−0.05	0.44^*^
Desmosterol	0.02	−0.35^*^	0.63^*^				−0.29	0.30	0.21
Brassicasterol	−0.87^*^	−0.33^*^	0.03				−0.07	0.15	0.29
Ergosterol	0.16	−0.31	−0.59^*^				−0.24	−0.05	−0.11
Campesterol	−0.17	0.45^*^	0.53^*^				0.11	−0.01	−0.05
Fucosterol	0.49^*^	−0.32^*^	−0.10				0.12	0.19	0.25
Stigmasterol	−0.32^*^	0.59^*^	−0.22				0.34^*^	0.08	−0.04
Fungisterol	0.56^*^	−0.24	0.11				−0.02	0.12	0.26
Corpisterol	0.15	−0.28	−0.54^*^				0.06	−0.01	0.20
β-Sitosterol	−0.07	0.77^*^	0.19				0.40^*^	0.13	−0.03
Chondrillasterol	0.57^*^	−0.24	0.12				0.03	0.12	0.34^*^
Schottenol	0.59^*^	−0.22	0.11				0.01	−0.10	0.36^*^
14:0				0.32^*^	0.43^*^	−0.46^*^	−0.29	0.37^*^	−0.53^*^
iso−15:0				−0.07	−0.46^*^	−0.05	0.08	−0.50^*^	−0.06
16:0				−0.30	−0.91^*^	0.11	0.25	−0.93^*^	0.09
16:1ω13				0.12	0.07	−0.50^*^	−0.12	0.03	−0.52^*^
16:1ω7				0.90^*^	−0.20	−0.25	−0.91^*^	−0.20	−0.23
16:2ω6				−0.43^*^	0.03	0.00	0.42^*^	0.02	0.03
16:2ω5/4				0.58^*^	0.14	0.07	−0.58^*^	0.15	0.09
16:3ω4				0.56^*^	0.13	0.11	−0.56^*^	0.13	0.13
18:1ω9				−0.43^*^	0.14	0.26	0.42^*^	0.16	0.28
18:1ω7				−0.10	0.01	−0.51^*^	0.10	−0.01	−0.54^*^
18:2ω6				−0.54^*^	0.25	−0.06	0.53^*^	0.23	−0.06
18:3ω3				−0.86^*^	0.11	−0.38^*^	0.85^*^	0.09	−0.41^*^
20:3ω6				0.05	0.42^*^	0.24	−0.03	0.42^*^	0.20
20:4ω6				0.44^*^	0.10	0.28	−0.44^*^	0.11	0.26
20:5ω3				0.53^*^	0.37^*^	0.53^*^	−0.54^*^	0.40^*^	0.50^*^
22:5ω6				−0.03	0.45^*^	0.17	0.05	0.44^*^	0.13
22:5ω3				0.50^*^	0.13	0.15	−0.50^*^	0.14	0.16
22:6ω3				−0.01	0.19	0.60^*^	0.02	0.21	0.56^*^
24:0				−0.41^*^	0.19	0.18	−0.41^*^	0.19	0.18

*Statistically significant correlations are marked with stars*.

NMS 1 of fatty acid data correlated negatively (*p* < 0.05, *r* > −0.56) with the fatty acids (16:1ω7, 16:2ω4, 16:3ω4, 20:5ω3 = EPA = eicosapentaenoic acid) typical in Diatomophyceae, but positively (*p* < 0.05, *r* > 0.46) with those typical in Chlorophyceae (16:2ω6, 18:3ω3). NMS 2 correlated positively (*p* < 0.05, *r* > 0.42) with the fatty acids (22:3ω6, 22:5ω6) typical in Synurophyceae and Cryptophyceae, but negatively (*p* < 0.05, *r* > -0.91) with 16:0 that is common in all algae. NMS 3 correlated positively (*p* < 0.05, *r* > 0.53) with EPA and DHA (docosahexaenoic acid, 22:6ω3) which are both common in Dinophyceae, and negatively (*p* < 0.05, *r* > -0.46) with 14:0, 16:1ω13, and 18:1ω7.

NMS of the combined data did not differ from the NMS ordination of the fatty acid data, and axes 1, 2, and 3 correlated with the same fatty acids as in the fatty acid data alone (Table 6). The only difference was that NMS 3 correlated positively (*r* > 0.44, *p* < 0.05) also with cholesterol.

## Discussion

Previous studies of the fatty acids of freshwater phytoplankton has shown separation at the class level (Taipale et al., 2013), but the sterol composition of different freshwater phytoplankton taxa has not been previously studied systematically using multivariate statistical analysis. Our analysis of 37 dominant freshwater phytoplankton species from 10 classes showed that even though both fatty acids and sterols varied significantly among phytoplankton classes, the integrity of sterol signatures within classes was lower than in fatty acids. The combined dataset of sterols and fatty acids showed similar multivariate dispersion to the fatty acid data (Figure 3), but in some cases mean distance-to-centroids among class decreased with the addition of sterol data (Figure 4). Phytoplankton class explained ~70% of the variation in fatty acid signatures and combined fatty acid and sterol signatures when Cyanophyceae were excluded. The percentage decreased 20% when Cyanophyceae were included in the fatty acid or the combined data (Table 4). While fatty acid profiles of phytoplankton are characteristic at class level, sterols seem to give information of the genus level for some phytoplankton classes.

The average numbers of detected fatty acids and sterols from individual freshwater phytoplankton strain were 19 and 4, respectively. Rampen et al. (2010) found 44 sterols in 106 marine diatoms, of which 15 were abundant and the rest were only found in less than 3% of their strains. In addition to phytoplankton, in many other organisms only one or a few sterols dominated the profile (Goad and Akhisa, 1997; Martin-Creuzburg and Von Elert, 2009). While the absence or presence of one fatty acid has a minor role in dissimilarities among taxa, a difference of one sterol could result in high dissimilarity in multivariate statistics. Therefore, we also investigated the combined data of fatty acids and sterols. We found that the contribution of sterols to total biomass of fatty acids and sterols was generally low and thus sterols had a minor role in separating phytoplankton classes. In NMS analysis (Figure 3) cholesterol was the only sterol correlating with any of the three NMS dimensions, and it correlated positively together with EPA and DHA with the NMS 3 that was responsible for separating Dinophyceae from other phytoplankton classes.

The multivariate analysis and Bayesian mixing modeling (Strandberg et al., 2015) separate different phytoplankton phylogenetical groups based on similarities and dissimilarities in biomolecule profiles. Therefore, models do not emphasize specific single biomarkers, and thus e.g. dinosterol, was not a significant component for separating Dinophyceae from other phytoplankton groups. It should also be remembered that even if phytoplankton are lacking some of the fatty acids and sterols characteristic to other phytoplankton groups, NMS clusters samples using similarities based on the whole profiles. For example *Aphanothece* and *Synechococcus* did not contain any sterols, or ω-3 or ω-6 fatty acids, but NMS clustered them together with Diatomophyceae due to their high 16:1ω7 content in these cyanophyceael strains. Therefore, for better separation of Cyanophyceae from algae more Cyanophyceae and other phytoplankton taxa should be analyzed for sterols and fatty acids. Also, a higher number of studied strains in all classes would give better separation and more detailed understanding on the relationship between sterols and phytoplankton phylogeny.

### Suitability of lipid biomolecules of metabolic pathways for biomarkers

Specific chemotaxonomic fatty acids or sterols for phytoplankton taxa are often by-products of metabolic pathways. For example, the polyunsaturated fatty acids 16:2ω6 and 16:3ω3 are precursors of 16:4ω3 during the ω3 desaturation pathway from 16:1ω9 (Erwin, 1973), and all of these fatty acids are found in Chlorophyceae, Conjugatophyceae, and Trebouxiophyceae. Furthermore, Diatomophyceae contain Δ^6^ desaturase activity that can desaturate 16:1ω9 to16:2ω4 and 16:3ω4, but 16ω3 PUFA are not found in Diatomophyceae (Perkins and Witting, 1975). Both ω3 and ω6 C_18_ PUFA are synthesized from oleic acid via desaturation and elongation. SDA (stearidonic acid, 18:4ω3) is synthesized from ALA (α-linolenic acid, 18:3ω3) by desaturation, but Dinophyceae prefer to retroconvert EPA to 18:5ω3 than desaturate SDA to 18:5ω3 (Perkins and Witting, 1975). However, whereas marine and brackish water dinoflagellates have been reported to have 18:5ω3 and DHA as their major FA (Leblond et al., 2006; Mooney et al., 2007), we found only trace amounts of 18:5ω3 in Dinophyceae, and more SDA and ALA. We also found higher amounts of EPA especially in *Peridinium cintum* than in marine or brackish water Dinophyceae strains, and *Peridinium* did not contain any 18:5ω3, indicating that the higher EPA content can be the result of lower retroconversion activity.

Among phytoplankton, the Cyanophyceae is the most diverse phytoplankton class according their FA profiles (Figures 3, 4), and especially their ability to desaturate oleic acid (18:1ω9) to C_18_ PUFA varies greatly between distinct genera, which have been used in previous studies in classifying Cyanophyceae into four groups (Kenyon, 1972, Murata et al., 1992, Los and Mironov, 2015). The cultured Cyanophyceae, *Aphanothece*, and *Synechococcus*, lacked C_18_ PUFA, but contained high amounts of C_16_ MUFA (monounsaturated fatty acid), and thus they belong to the first group. Additionally, even though *Phormidium* was able to synthesize C_18_ PUFA, we found only limited amount of 18 PUFA in this genus. The second group of Cyanophyceae has the first double bond of triunsaturated C_18_ PUFA at the position ω3 and group 3 at the position ω6. The only genus of cultured Cyanophyceae in our study that belonged to group 2 was *Microcystis*, whereas the rest of the cultured strains (*Snowella, Anabaena, Planktothrix, Limnothrix, Pseudanabaena*) belonged to group 3. The fourth group had four acyl-lipid fatty acid desaturases and can synthesize SDA (Los and Mironov, 2015). However, none of our cyanobacterial strains belonged to this group.

Algal sterol biosynthesis is much more complicated than that of fatty acids, having more steps toward the final product and resulting in many subproducts and a high number of different phytosterols (Nes and McKean, 1977). Algal sterols are synthesized via the mevalonate (MVA) or methyl-D-erythritol 4-phosphate (MEP) pathways of isoprenoid biosynthesis, and the synthesis requires approximately 30 different enzymes. Our goal was to analyze the major phytosterols in phytoplankton but not to analyze all sterols existing in trace amounts that are the precursors of some other sterols. Chemotaxonomical differences in synthesized sterols were found among classes even though some classes did not differ statistically from each other. *Chlamydomonas* has the ability to synthesize egrosterol and corbisterol from cycloartenol that differs from the fungal acetate-mevalonate pathway (Miller et al., 2012). In addition to *Chlamydomonas* we found high amounts of ergosterol and corbisterol in *Euglena*. Ergosterol and corbisterol (double bond positions at Δ^5, 7, 22^) have been previously found in some other marine Chlorophyceae genera (*Dunaliella, Polytoma, Haematococcus, Chlorella*) (Patterson, 1991). Unusual Δ^7^ and Δ^7, 22^ -sterols were only synthesized among Chlorophyceae in *Eudorina, Monoraphidium, Pediastrum, Acutodesmus*, and *Selenastrum*, which are the most common green algae in addition to *Chlamydomonas* in boreal lakes. Additionally, Δ^7^ and Δ^7, 22^ -sterols were the major sterols in *Closterium* and trace amounts of these sterols were found in *Cosmarium*, both members of Conjugatophyceae. Furthermore, other studies have found Δ^7^ and Δ^7, 22^ -sterols in two other marine Chlorophyceae: *Ankistrodesmus* and *Oocystis* (Patterson, 1991), that are also found in freshwater systems. Most dinoflagellates contain high amounts of 4α-methyl sterols, which are rare among other phytoplankton taxa (Mansour et al., 1999; Volkman, 2003). Among the two cultured Dinophyceae, *Peridinium* had more 4α-methyl sterols than *Ceratium*, which contained <5% of 4α-methyl sterols. Altogether, it could be concluded that by including only the fatty acids or sterols synthesized through pathways common only in one phytoplankton group, like here in Dinophyceae, the discrimination among classes can be enhanced in multivariate analysis.

### Sterols and fatty acids as chemotaxonomic biomarkers

Biomolecules can be useful biomarkers in food web studies for separating phytoplankton, terrestrial matter and bacteria, and to quantify their importance in the diets of zooplankton. Fatty acids have already been used for defining phytoplankton composition (Strandberg et al., 2015), but also for dietary analysis of zooplankton, fish and seals (Sargent et al., 1987; Iverson et al., 1997; Galloway et al., 2014). This is possible since the fatty acid analysis of freshwater phytoplankton and marine macrophytes has shown that the variation in fatty acid profiles is largely explained by taxonomic identity, and thus it seems that environmental conditions such as nutrients and light intensity have relatively lower impact on fatty acid composition (Galloway et al., 2012; Taipale et al., 2013; Bi et al., 2014; Galloway and Winder, 2015). Sterols can be used only for phytoplankton community analysis and not for trophic transfer since cholesterol is a major sterol in zooplankton and fish, is a precursor for steroid hormones (Grieneisen, 1994), and is a required for developmental patterning of animal embryonic structures (Porter et al., 1996).

Our PERMANOVA analysis showed that 50% of the variation in phytoplankton sterol composition was explained by class, and thus the major part of sterol variation was explained by phylogeny. Environmental conditions may affect the concentrations of individual sterols, and for example light, phosphorus, and temperature influence the abundance of Δ^7^-sterols and Δ^7, 22^-sterols (fungisterol, chondrillasterol and 22-dihydrochondrillassterol) in *Scenedesmus* (Veron et al., 1996; Piepho et al., 2010, 2012). In these studies *Scenedesmus* did not synthesize Δ^5^-sterols under any conditions, however, the growth phase influenced dinoflagellates to synthesize different sterols (Amo et al., 2010), thus showing some flexibility in sterol synthesis in freshwater algae.

The traditional biomarker approach is looking for specific biomolecules that are absent or rare in other organisms, but as mentioned above present multivariate models do not require specific biomarkers. Nevertheless, these specific biomarkers can be used to track biomass of specific phytoplankton class or genus. Previous studies have identified dinosterol and related sterols as unique biomarkers for dinoflagellates (Volkman et al., 1993; Piretti et al., 1997). Additionally, our previous study (Taipale et al., 2013) of freshwater phytoplankton showed C_15_ to C_17_ PUFA to be specific and useful biomarkers for Chlorophyceae and Trebouxiophyceae (16:2ω6, 16:3ω3 and 16:4ω3), Diatomophyceae (16:3ω4), and Euglenophyceae (C_15_ and C_17_ PUFA). However, simultaneous monitoring of fatty acids and sterols can provide more detailed information of phylogeny but also of nutritional adequacy. The simultaneous presence of 16ω3 PUFAs and ergosterol would indicate *Chlamydomonas* whereas Δ^5, 22^-sterols would point to *Sphaerocystis*, and Δ^7^-sterols to other genera of Chlorophyceae and Conjugatophyceae. Another useful approach could be to monitor 4α-desmethyl sterols simultaneously with DHA for tracking the abundance of Dinophyceae; this could also provide information on nutritional quality since DHA is physiologically the most important FA for copepod zooplankton and fish.

Previous studies have shown that sterol-coding genes (Villanueva et al., 2014) and 18S rRNA sequences together with sterol composition of marine diatoms (Rampen et al., 2010) can be used for forming algal groups. However, presently it's not possible to use same primers for all phytoplankton classes (including Cyanophyceae and microalgae), and thus we were forced to rely on present taxonomy. Phytoplankton taxonomy is based more on morphology than on gene sequencing, and therefore taxonomy relies on subjective opinions and present consensus (Reynolds et al., 2002). Thus, dissimilarities in sterol composition within present taxa do not necessarily mean an inconsistent pattern of sterol synthesis, but may indicate problems in species identification and classification. Of the strains we used, *Botryococcus* is placed under the Chlorophyceae in some classifications (Christensen, 1980), but gene sequencing separated it into the Trebouxiophyceae (Senoysu et al., 2004). Our sterol analysis showed that *Botryococcus* together with *Sphaerocystis* has Δ^5^-sterols, and thus these genera together with *Chlamydomonas* differ chemotaxonomically from the other green algae (Pröschold et al., 2001). However, the somatic growth and reproduction of herbivorous zooplankton was similar with *Acutodesmus* and *Chlamydomonas* diets, indicating nutritional similarity in spite of difference in sterol composition (Taipale et al., 2014). More studies of biochemical analysis of different freshwater strains together with molecular tools would be of utmost importance for more precise phytoplankton chemotaxonomy and the true phylogeny.

Multivariate statistics do not only reveal chemotaxonomic relationship of freshwater phytoplankton, but also can reveal larger groups which are able to synthesize essential fatty acids or sterols. The abundance of ALA, EPA, and DHA are important diet components defining food quality for herbivorous zooplankton and the whole food web. In our analysis NMS axis 3 (Figure 3) correlated strongly with DHA thus revealing taxa which can synthesize DHA: Dinophyceae, Diotomophyceae, Cryptophyceae, and Chrysophyceae. For sterols the dietary difference is not so clear, but in laboratory studies Martin-Creuzburg et al. (2014) showed that fucosterol, brassicasterol, ergosterol, and stigmasterol supported the somatic growth of *Daphnia* better than cholesterol, lathosterol, cholestanol, or 7-dehydrocholesterol, thus green algae (Chlorophyceae, Conjugatophyceae, Figure 3) are a potentially weaker quality diet for herbivorous zoonplankton. This is mostly the case in eutrophic lakes, since green algae are not so abundant in other lake types of the boreal zone (Lepistö and Rosenström, 1998). However, more studies with zooplankton are required.

Altogether, our conclusion is that fatty acid and sterol composition of freshwater phytoplankton differs at class level with some dissimilarity in sterols profiles. Actually, whereas fatty acid profiles can be used for defining phytoplankton composition at class level, additional sterol analysis can tell genus level composition in some cases. For example, ergosterol might be an excellent biomarker for *Chlamydomonas* in small boreal lakes to discriminate between different Chlorophyceae, where *Euglena* is usually absent. Additionally, 4α-methyl sterols can be used to track Dinophyceae, but also potentially to distinguish between *Peridinium* and *Ceratium* whose 4α-methyl sterols differed from each other. The usability of the fatty acid-based Bayesian mixing modeling (e.g., FASTAR, Fatty Acid Source Tracking Algorithm in R; Strandberg et al., 2015) could be improved by adding sterols, but for that more sterols profiles of freshwater strains should be analyzed.

## Author contributions

ST, EP, and KV designed the study. ST, EP, and KV cultured different phytoplankton strains and ST extracted and analyzed sterols and fatty acids from phytoplankton. KV calculated major phytoplankton composition using phytoplankton database of Finnish Environment Institute. ST and MH did statistical analysis. ST wrote the paper. All authors discussed the results and commented on the manuscript.

### Conflict of interest statement

The authors declare that the research was conducted in the absence of any commercial or financial relationships that could be construed as a potential conflict of interest.
